# Cloning and characterization of a novel cold-active glycoside hydrolase family 1 enzyme with β-glucosidase, β-fucosidase and β-galactosidase activities

**DOI:** 10.1186/1472-6750-13-22

**Published:** 2013-03-15

**Authors:** Anna Wierzbicka-Woś, Paulina Bartasun, Hubert Cieśliński, Józef Kur

**Affiliations:** 1 Department of Microbiology, Faculty of Biology, University of Szczecin, Felczaka 3c, Szczecin 71-412, Poland; 2 Department of Microbiology, Gdańsk University of Technology, Narutowicza 11/12, Gdańsk 80-233, Poland

**Keywords:** Metagenomics, Cold-active enzyme, β-galactosidase, β-glucosidase, β-fucosidase

## Abstract

**Background:**

Cold-active enzymes, sourced from cold-adapted organisms, are characterized by high catalytic efficiencies at low temperatures compared with their mesophilic counterparts, which have poor activity. This property makes them advantageous for biotechnology applications as it: (i) saves energy costs, (ii) shortens the times for processes operated at low temperatures, (iii) protects thermosensitive substrates or products of the enzymatic reaction, (iv) prevents undesired chemical transformations, and (v) prevents the loss of volatile compounds.

**Results:**

A *bglMKg* gene that encodes a monomeric cold-active glycoside hydrolase family 1 enzyme with an apparent molecular mass of 50 kDa was isolated by the functional screening of a marine metagenomic library. The BglMKg enzyme was expressed in *E. coli,* purified by FPLC and characterized. The recombinant BglMKg could effectively hydrolyze various chromogenic substrates and β-linked oligosaccharides, and had remarkably high β-galactosidase, β-glucosidase and β-fucosidase activities. Because of the lack of information about the usefulness of β-fucosidases in industry, further characterization of the enzymatic properties of BglMKg was only carried out with substrates specific for β-glucosidase or β-galactosidase. The BglMKg had maximal β-galactosidase and β-glucosidase activities at approximately 40°C and 45°C, respectively. The optimum pH for β-galactosidase activity was 6.5, whereas the optimum pH for β-glucosidase activity was 7.5. In general, the enzyme was stable below 30°C and from pHs 6.0 to 8.0. The results of the kinetic studies revealed that BglMKg more efficiently hydrolyzed β-glucosidase substrates than β-galactosidase ones.

**Conclusions:**

BglMKg is a small, monomeric, cold-active β-glucosidase with additional enzymatic activities. It was efficiently expressed in *E. coli* indicating that BglMKg might be a candidate for industrial applications.

## Background

Metagenomics, the analysis of DNA isolated from environmental samples, has proven particularly useful for the study of uncultured bacteria, given that it has been estimated that less than 1% of microorganisms found in natural environments can be cultured with currently available technologies [1]. In light of the enormous abundance of uncultivated microbes that are adapted to a wide range of physical-chemical parameters matching industrial requirements with regards to pH, pressure and temperature, metagenomics potentially opens the door to new sources of industrial enzymes with unique properties [2-4]. In this context, the psychrophilic and some psychrotrophic microorganisms, which are capable of thriving in cold environments, seem to be an excellent source of enzymes characterized by high catalytic rates at low temperatures. In general, a low-temperature industrial process saves energy, protects the thermolabile substrates and/or products from degradation, and decreases the rate of the nonspecific chemical reaction. Moreover, in the food industry, a low-temperature enzymatic process could decrease the risk of infection by, and growth of, mesophilic microorganisms, especially those pathogenic for human and animals.

β-Galactosidases (E.C. 3.2.1.23) have been extensively studied for their utility in a variety of industrial technologies. In general, β-galactosidases catalyze the hydrolysis of the O-glycosidic bond in β-galactosides, such as lactose. At present, a commercially available β-galactosidase from the mesophilic yeast *Kluyveromyces lactis* is used in the production of lactose-reduced milk for people with lactose intolerance. In addition, the hydrolysis of lactose in dairy products increases their sweetness and eliminates the ‘sandy defect’ arising during lactose crystallization at low temperatures [5,6]. However, the main disadvantage of this mesophilic enzyme as an industrial biocatalyst is its poor activity at temperatures below 20°C. Ideally, a β-galactosidase for treating refrigerated milk in the dairy industry should be highly active and stable at approximately 10°C and easy to inactivate at a higher temperature. Moreover, an enzyme of this nature should be active and stable at pH 6.7-6.8, and not be inhibited by ions or monosugars, which are natural products of lactose hydrolysis, such as Ca^2+^, or d-glucose and d-galactose, respectively. Therefore, a great deal of effort has been invested in the isolation and characterization of new cold-active β-galactosidases from cultivable, cold-adapted bacteria and yeasts [7]. However, to the best of our knowledge, a cold-adapted β-galactosidase, which would satisfactorily fulfill the abovementioned requirements and, at the same time, be easy and inexpensive to manufacture, has not yet been identified.

Our previous studies focused on the identification and characterization of cold-active β-galactosidases that were sourced from culturable bacterial strains [7-10]. Therefore, in this study, we decide to apply the metagenomic approach, which could also serve to expand our search for β-galactosidases derived from nonculturable bacteria. To this end, a plasmid metagenomic DNA library was constructed using total DNA isolated from a Baltic Sea water sample. Through activity-based screening of the resulting DNA library for β-galactosidase active clones, a novel glycoside hydrolase gene, designated as *bglMKg* was isolated. The *bglMKg* gene was cloned, expressed in *Escherichia coli*, and the detailed biochemical characterization of the recombinant enzyme was conducted.

## Results and discussion

### Construction of a metagenomic library and screening for clones encoding β-galactosidase activity

To isolate the gene encoding the enzyme with β-galactosidase activity, a metagenomic library containing about 1100 clones was constructed using DNA obtained from a sample of Baltic Sea water collected in Kołobrzeg, Poland. After 48 h incubation of recombinant *E. coli* colonies at 20°C, only one colony turned blue on LB plates supplemented with 5-bromo-4-chloro-indolyl-β-d-galactopyranoside (X-gal). This positive clone with β-galactosidase activity, designated as insMKg, was selected for further characterization. The DNA of the recombinant plasmid pBAD/insMKg was extracted and digested with selected restriction enzymes in order to create restriction maps of the construct.

### The DNA sequence analysis of a metagenomic DNA insert of pBAD/insMKg

**Figure 1:**
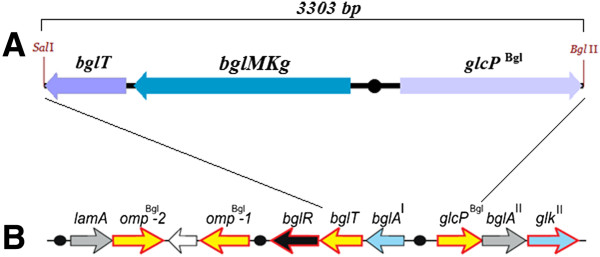
**The presence of open reading frames (ORF) in the metagenomic insert (A) and how they correspond with the arrangement of the β-glucoside utilization pathway from *Shewanella amazonensis *(B); *bglA***^***I***^** – gene encoding the putative cytoplasmic β-glucosidase, *bglT, omp***^***Bgl***^** – genes encoding the putative β-glucosides transporters,* glcP***^**Bgl**^** – gene encoding the putative β-glucose permease, *bglA***^**II**^** – gene encoding the putative periplasmatic β-glucosidase,* glk***^**II**^** – gene encoding the putative ROK-type glucokinase, *lamA* – gene encoding the extracellular β-glucanase, *bglR* – gene encoding the putative novel LacI-type transcriptional regulator; the black circles represent the predicted binding sequences for the putative transcriptional regulators of the Bgl utilization pathway (Rodionov D.A. et al. 2010).**

The nucleotide sequence analysis revealed that the metagenomic DNA insert of the pBAD/insMKg plasmid contained two partial ORFs at the 5^′^ and 3^′^ terminals and a complete ORF in the middle (Figure 1A). The partial ORF1, the complete ORF2, and the partial ORF3 revealed the highest sequence homology to the DNA sequences of the *Sfri_1317*, *Sfri_131*6 and *Sfri_1315* genes from *Shewanella frigidimarina* NCIMB 400 (GenBank: NC_008345.1), respectively. Moreover, the layout of the ORFs from the metagenomic DNA insert corresponded to the layout of the *Sfri_1317, Sfri_1316 and Sfri_1315* genes in the genome of *Shewanella frigidimarina* NCIMB 400. Further analysis revealed that the layout of these three ORFs also corresponded to the layout of three genes from the Bgl cluster genes encoded proteins involved in the putative β-glucoside-containing glucans utilization pathway in *Shewanella* spp. (Figure 1B) [11]. Moreover, this comparative sequence analysis also revealed that the partial ORF1 and the partial ORF3 corresponded to the *bglT* and *glcP*^Bgl^ genes of the Bgl cluster, respectively. The *bglT* and *glcP*^Bgl^ genes encoded a putative glucose/galactose transporter and a putative sugar (glycoside-pentoside-hexuronide) transporter, respectively, whereas the ORF2 corresponded to the *bglA*^I^ gene, and encoded one of three putative glucosidases of the reconstructed Bgl utilization pathway in *Shewanella* spp. Rodionov *et al.* proposed that two putative glucosidases, LamA and BglA^II^, are secreted outside of the cell and to the periplasm, respectively, whereas BglA^I^ is most likely a cytoplasmic enzyme. The extracellular endo-β-1,3-glucanase LamA hydrolyses β-glucoside-containing glucans to oligo-β-glucosides, which are transported to the periplasm by Omp^Bgl^, and subsequently utilized by BglA^II^ to produce d-glucose and shorter β-glucosides. Finally, the products of hydrolysis, such as cellobiose or gentiobiose, are taken up by the predicted BglT transporter into the cytoplasm, where they are finally hydrolyzed by the BglA^I^ enzyme [11]. In light of this data, it seemed possible that the ORF2, named *bglMKg*, encoded an enzyme with β-glucosidase activity specific toward disaccharides consisting mainly of two glucose molecules. Therefore, we also tested this hypothesis during the study.

The nucleotide sequence analysis of the metagenomic DNA insert also identified the putative promoter sequences: (*i*) 460 bp upstream a *bglMKg* gene, predicted with the BProm program, and (*ii*) 154 bp or 90 bp upstream the *bglMKg* gene, predicted with the Neural Network Promoter Prediction program. Therefore, in our opinion, it appears possible that the *bglMKg* gene was expressed from its own promoter in *E. coli* cells (the recombinant clone insMKg). Moreover, we discovered that the consensus DNA-binding motif proposed by Rodionov *et al.*[11] as a binding site for a LacI-type transcriptional regulator BglR is present 197 bp upstream from the *bglMKg* gene (Figure 1A and 1B).

### Sequence analysis of BglMKg

The deduced product of *bglMKg* consists of 442 amino acid residues with a calculated molecular mass of 50,133 Da and shares a highest sequence identity of 75% with a β-glucosidase encoded by the *Sfri_1316* gene from *Shewanella frigidimarina* NCIMB 400 (NCBI Reference Sequence: YP_750007.1). However, to the best of our knowledge, the putative β-glucosidase of *Shewanella frigidimarina* NCIMB 400 has not been biochemically characterized to date. In contrast, among the well-characterized enzymes deposited in the GenBank database (GenBank No.: GU647096), the highest sequence identity (65%) to the BglMKg enzyme was shown by a metagenomic-derivate Bgl1A β-glucosidase [12]. Interestingly, the Bgl1A enzyme revealed a lack of β-galactosidase activity.

Prediction of the occurrence of functional domains and a putative active site in BglMKg with InterProScan allowed the BglMKg enzyme to be classified as a new member of Glycoside Hydrolase Family 1 (abr. GH1) [13]. GH1 comprises enzymes with a number of known activities, such as, β-glucosidase (EC 3.2.1.21); β-galactosidase (EC 3.2.1.23); β-mannosidase (EC 3.2.1.25); β-glucuronidase (EC 3.2.1.31); β-fucosidase (EC 3.2.1.38); 6-phospho-β-galactosidase (EC 3.2.1.85); and 6-phospho-β-glucosidase (EC 3.2.1.86) (http://www.cazy.org/Glycoside-Hydrolases.html). Moreover, this analysis revealed that residues E169 and E352 of BglMKg correspond to the conserved catalytic glutamic acid residues involved in the catalytic activity of GH1 enzymes (Pfam method).

On the other hand, the similarity of the BglMKg enzyme to the BglA^I^ β-glucosidase proposed as a cytoplasmic enzyme by Rodionov *et al.*[11], encouraged us to examine the possibility that BglMKg is subcellularly localized. Therefore, owning to the high sequence similarity of ORFs in the metagenomic DNA insert to the *Sfri_1317, Sfri_1316* and *Sfri_1315* genes in the genome of *Shewanella frigidimarina* NCIMB 400, we assumed that the our metagenomic DNA is also of bacterial origin. The analysis of the BglMKg amino acid sequence with the SignalP 4.0 server in relation to this assumption revealed the lack of any signal sequence in BglMKg that could be involved in its transport to the periplasmic space or outside the bacterial cell. Moreover, another sequence analysis, carried out with the PROSITE program, showed the localization of the BglMKg enzyme in the cytoplasm. In short, the results presented here suggest that the BglMKg enzyme could be a cytoplasmic enzyme of bacterial origin.

### Expression and purification of the BglMKg enzyme

**Figure 2:**
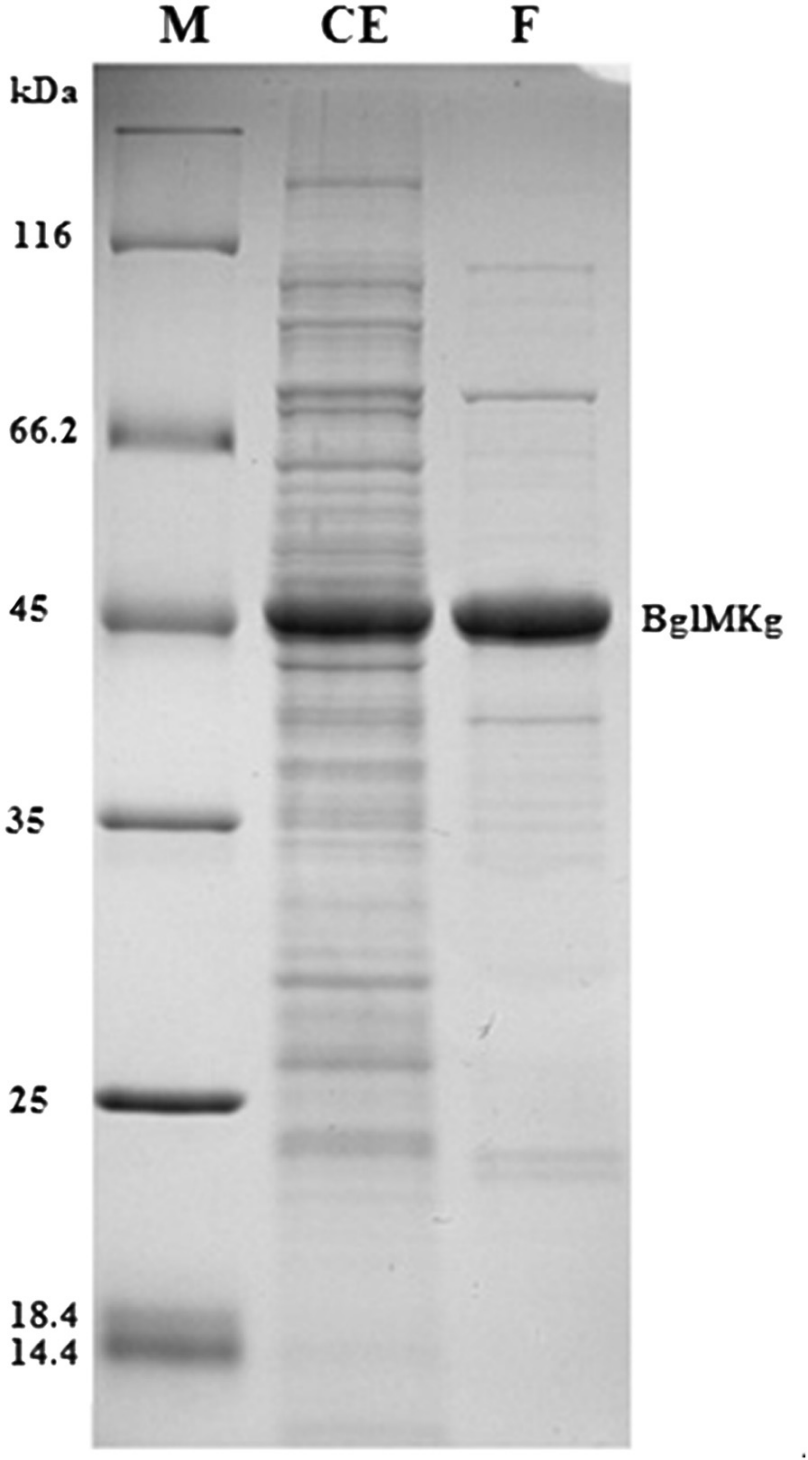
**SDS-PAGE protein profiles of fractions collected during purification of recombinant BglMKg enzyme.** Lanes: M – protein weight marker, CE – cell extract after expression, F – purified fraction of enzyme BglMKg after purification on Fractogel EMD DEAE.

The arabinose-inducible promoter of the pBAD-Myc-His A plasmid was used for the expression of the metagenomic-derived *bglMKg* gene in *E. coli* LMG194 cells. The highest enzyme production yields were achieved by adding l-arabinose to a final concentration of 0.2% w/v, at A_600_ OD = 0.55 and by further cultivation at 30°C for 8 h. The recombinant BglMKg protein was purified using FPLC and the procedure is summarized in Table 1. The obtained enzyme was ~85% pure (densitometric analysis; software ImageJ v 1.44I) as determined by SDS-PAGE (Figure 2). Unfortunately, we found that the further purification of BglMKg with the Resource Q column led to a remarkable decrease of approximately 50% in enzyme activity, without significantly increasing the purity (purification fold = 0.4). Therefore, we decided to reduce the purification procedure to the one-step.

**Table 1:** Purification of the recombinant BglMKg (U for β-galactosidase activity)

**Step**	**Total protein (mg)**	**Total activity (U)**	**Specific activity (U/mg)**	**Purification (fold)**	**Yield (%)**
Crude extract	434.5	759	1.75	1.0	100
Fractogel EMD DEAE	72.0	302	4.19	2.4	40

The enzyme had an estimated apparent molecular weight of approximately 50 kDa corresponding to the expected molecular weight calculated from the BglMKg amino acid sequence. The relative molecular mass of recombinant BglMKg, which was determined by gel filtration, was ~50 kDa, suggesting that the native enzyme is a monomer protein. To date, several other enzymes belonging to GH1 family have also been reported as monomeric proteins [14,15].

### Substrate specificity of BglMKg

Owing to the variety of enzymatic activities within glycoside hydrolases family 1, we decided to examine the enzymatic specificity of BglMKg toward the various chromogenic substrates presented in Table 2. The enzyme hydrolyzed *p*-NP-β-d-glucopyranoside (β-glucosidase activity), *p*-NP-β-d-fucopyranoside (β-fucosidase activity), *o*-NP-β-d-galactopyranoside (β-galactosidase activity), *p*-NP-β-d-galactopyranoside (β-galactosidase activity), *p*-NP-β-d-cellobioside (exo-β-glucanase) and *p*-NP-β-d-xylopyranoside (β-xylanase). This is consistent with past studies, which have also reported GH1 enzymes with a wide range of enzymatic activities [15-17]. What is important to note is that the BglMKg enzyme revealed higher relative activities against substrates specific for β-glucosidase (130%) and β-fucosidase (133%) than against *o*-NP-β-d-galactopyranoside (refer as 100%) and *p*-NP-β-d-galactopyranoside (82%), the substrates specific for β-galactosidase. Moreover, the BglMKg showed markedly higher activity against the *p*-NP-β-d-glucopyranoside, an analogue of a cellobiose consisting of two glucose molecules linked by a β-1→4 bond, than it did for *p*-NP-β-d-cellobioside, an analogue of a cellotriose consisting of three glucose molecules linked by β-1→4 bonds, respectively.

**Table 2:** Relative activity of purified BglMKg enzyme with various nitrophenyl-derived chromogenic substrates

**NP-derived substrate**	**Relative activity (%)**
*o*-NP-β-d-galactopyranoside	100
*p*-NP-β-d-galactopyranoside	82
*p*-NP-β-d-glucopyranoside	130
*p*-NP-β-d-fucopyranoside	133
*p*-NP-β-d-cellobioside	59
*p*-NP-β-d-xylopyranoside	4
*p*-NP-β-d-galacturonide	< 0.01
*p*-NP-β-l-arabinopyranoside	< 0.01
*p*-NP-β-d-mannopyranoside	< 0.01
*p*-NP-β-d-glucuronide	< 0.01
*p*-NP-α-d-galactopyranoside	< 0.01

Moreover, we also determined the enzymatic activity of BglMKg against various disaccharides consisting of two glucose molecules linked by α- or β-glycosidic bonds and lactose consisting of one glucose molecule and one galactose molecule linked by a β-glycosidic bond, respectively. The results presented in Table 3 demonstrate that BglMKg revealed the highest relative enzymatic activity against cellobiose (100%; β-1→4 bond), compared with its relative activities against lactose (47%; β-1→4 bond), sophorose (44%; β-1→2 bond), and gentiobiose (7%, β-1→6 bond). Moreover, the enzymatic activity of BglMKg against substrates with different kinds of α-glycosidic bonds was not detected (Table 3). To summarize, in the light of the results presented herein, it seems highly probable that BglMKg is a cytosolic β-glucosidase with high enzymatic activity against disaccharides consisting primarily of glucose molecules linked by β-glycosidic bonds.

**Table 3:** Relative activity of purified BglMKg enzyme with various disaccharides

**Disaccharide**	**Relative activity (%)**
Cellobiose	100
Sophorose	44
Gentiobiose	7
Lactose	47
Nigerose	< 0.01
Trehalose	< 0.01
Maltose	< 0.01
Isomaltose	< 0.01

With regard to possible industrial applications, further characterization of the enzymatic properties of BglMKg was carried out using substrates specific for β-galactosidase (*o*-NP-β-d-galactopyranoside, ONPGal) and β-glucosidase (*p*-NP-β-d-glucopyranoside, PNPGlc). However, given the lack of information about the usefulness of β-fucosidases in industry, further characterization of BglMKg with substrate(−s) specific for β-fucosidase was not carried out. We determined the influence of the temperature, pH, and products of cellobiose or lactose hydrolysis on both the enzymatic activities of BglMKg, respectively.

### Physicochemical characterization and determination of kinetic parameters

**Figure 3:**
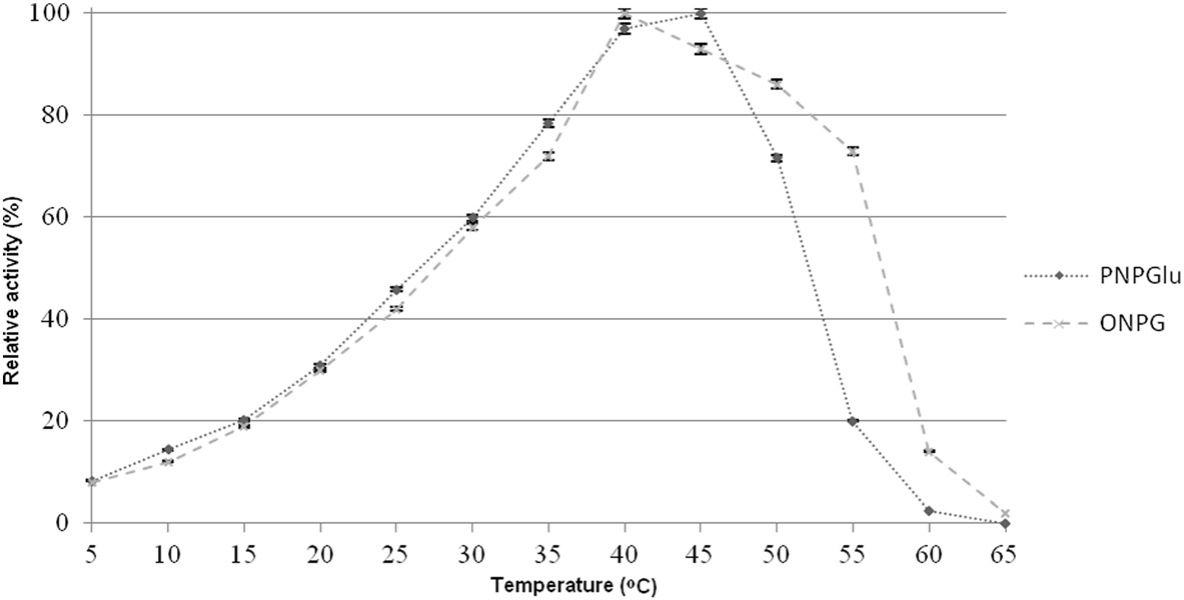
The effect of temperature on the recombinant BglMKg enzyme activities.

**Figure 4:**
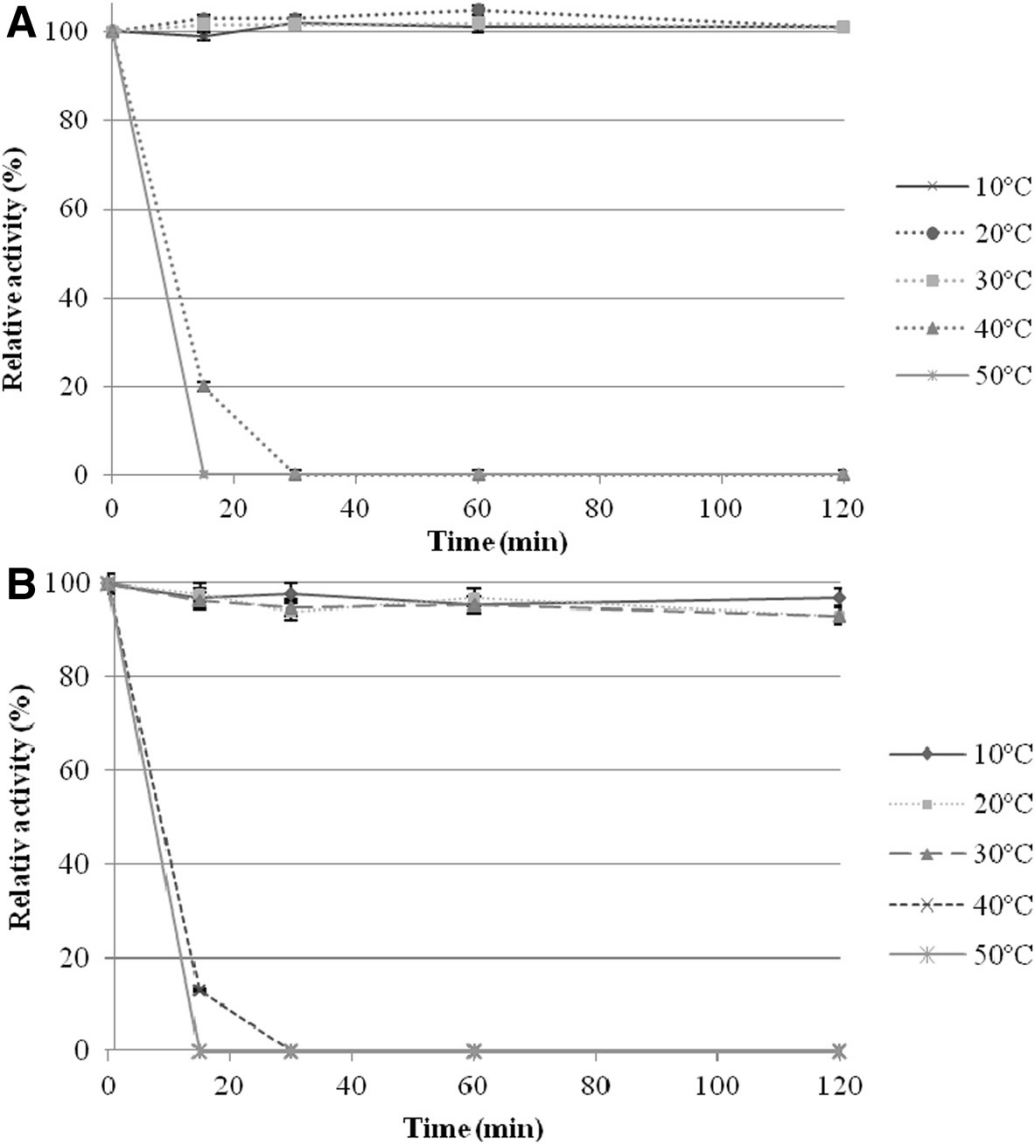
The effect of temperature on the recombinant BglMKg β-galactosidase stability (A), and β-glucosidase stability (B).

The optimal temperatures for the β-galactosidase and β-glucosidase activities of BglMKg were determined over a temperature range of 0°C to 65°C. As shown in Figure 3, BglMKg had almost the same relative activities for ONPGal and PNPGlc over a temperature range of 0°C to 40°C. Maximal β-galactosidase and β-glucosidase activities were observed at 40°C and 45°C, respectively. The relative activities of BglMKg above 50°C were higher for ONPGal than for PNPGlc (Figure 3). Moreover, we determined that both the enzyme activities were retained at 97% after 2 h of incubation over a temperature range of 10 to 30°C, and that the enzyme was rapidly inactivated at temperatures above 40°C (Figure 4A and 4B).

**Figure 5:**
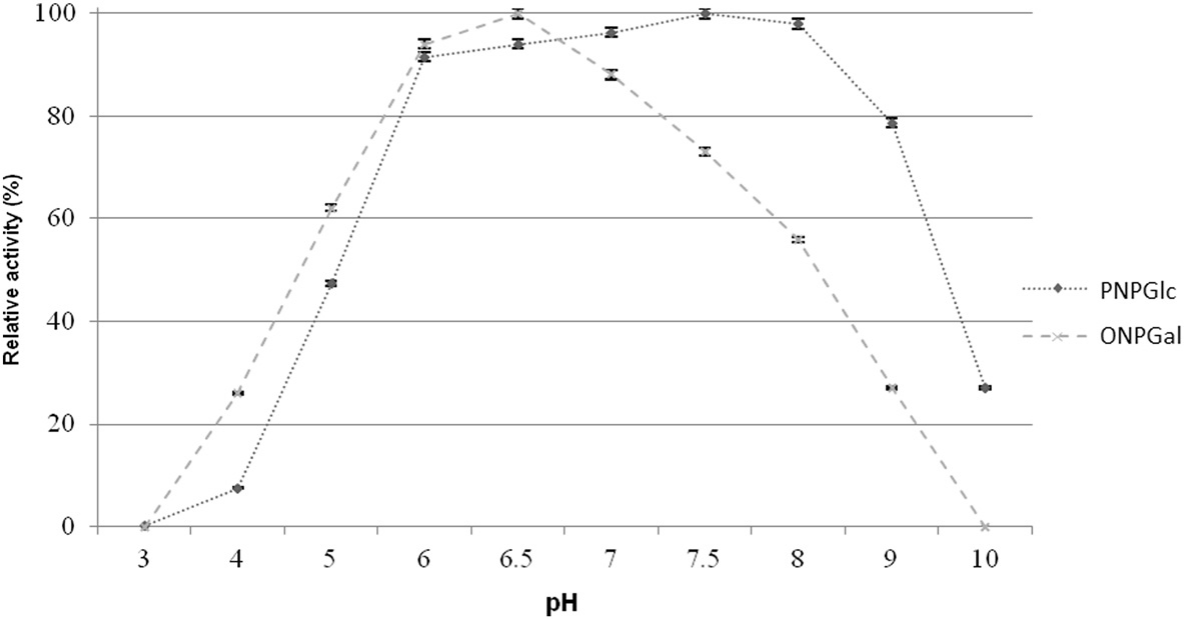
The effect of pH on the recombinant BglMKg enzyme activities.

**Figure 6:**
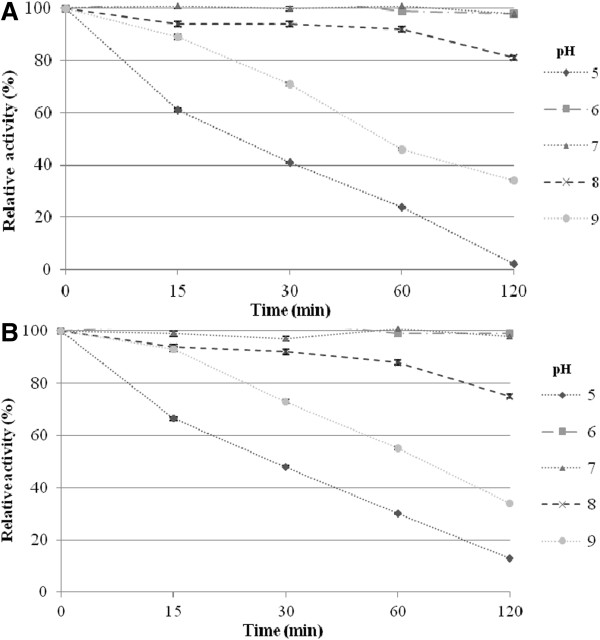
The effect of pH on the recombinant BglMKg β-galactosidase stability (A), and β-glucosidase stability (B).

The optimal pHs for the β-galactosidase and β-glucosidase activities of BglMKg were studied over a pH range of 3.0 to 11.0 and at 20°C. As shown in Figure 5, BglMKg with ONPGal as a substrate had above 90% of maximum activity at pH 6.0–7.0, with the maximum at pH 6.5. In contrast, with PNPGlc as a substrate, it had above 90% of maximum activity over a broad pH range of 6.0–8.5, with the maximum at pH 7.5. In addition, we found that both the enzyme activities remained at 98% from pH 6.0 to 7.0, and at ~80% at pH 8.0, after 2 h of incubation (Figure 6A and 6B). The enzyme was rapidly inactivated at pHs below 5.0 and above 9.0.

**Figure 7:**
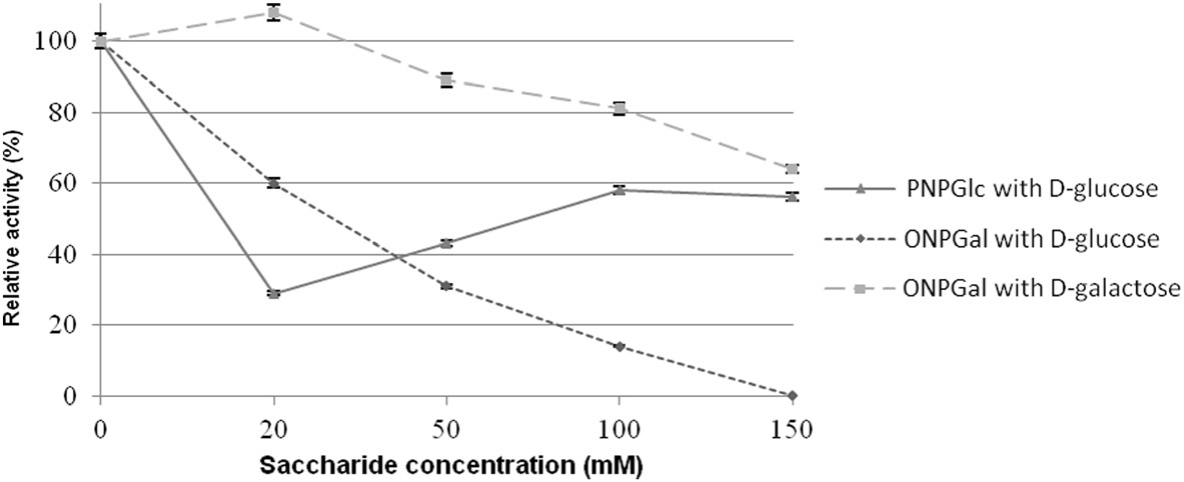
**The effects of the different concentrations of d-glucose and d-galactose on the recombinant BglMKg enzyme activities.**

The β-galactosidase activity of BglMKg toward ONPGal continually decreased with an increase of d-glucose from 20 mM to 150 mM, whereas it was slightly increased in the presence of d-galactose at 20 mM and decreased by d-galactose at 50, 100 and 150 mM (Figure 7). In contrast, the β-glucosidase activity of BglMKg was strongly inhibited by d-glucose at 20 mM, and less so at the higher concentrations of d-glucose (Figure 7).

The study of the kinetic properties (*K*_*m*_*, k*_*cat*_ and *k*_*cat*_*/K*_*m*_) revealed that BglMKg had higher affinities (*K*_*m*_ values) for cellobiose and PNPGlc than lactose and ONPGal (Table 4). Moreover, Table 4 shows that the *k*_*cat*_*/K*_*m*_ values for cellobiose and PNPGlc are approximately 10 times higher than the *k*_*cat*_*/K*_*m*_ values for lactose and ONPGal. These results indicate that BglMKg is much more efficient at the hydrolysis of β-glucosidase substrates than for the β-galactosidase ones.

**Table 4:** Kinetic parameters of BglMKg

**Substrate**	**Temperature (°C)**	***K***_***m ***_**(mM)**	***k***_***cat ***_**(s**^**-1**^**)**	***k***_***cat***_**/*K***_***m ***_**(s**^**-1**^**mM**^**-1**^**)**
	10	4.82 ± 1.10	3.99 ± 0.53	0.83
**ONPGal**	20	9.04 ± 1.39	12.43 ± 1.35	1.37
	30	5.72 ± 0.60	14.73 ± 0.95	2.57
	10	0.13 ± 0.06	4.46 ± 0.28	34.31
**PNPGlc**	20	0.16 ± 0.03	11.74 ± 0.26	73.37
	30	0.37 ± 0.04	22.45 ± 0.64	60.67
	10	73.89 ± 46.97	7.95 ± 3.99	0.11
**Lactose**	20	41.56 ±13.31	8.55 ±1.89	0.12
	30	86.91 ± 20.32	24.30 ±4.64	0.28
	10	3.55 ± 1.76	6.35 ± 0.7	1.79
**Cellobiose**	20	3.89 ± 0.98	10.46 ± 0.67	2.72
	30	2.76 ± 0.51	11.19 ± 0.43	4.05

In summary, the pH of natural milk is about 6.7–6.8, which could suggest that BglMKg is a suitable enzyme for the hydrolysis of lactose in refrigerated milk. However, in our opinion, the catalytic properties of this enzyme and its strong inhibition by d-glucose and d-galactose disqualify it as a new candidate for developing a low-cost, lactose hydrolysis technology based on an enzymatic process. In contrast, in the light of data presented here, the BglMKg enzyme appears to be an interesting industrial candidate as a novel cold-active β-glucosidase. First, at ~48 kDa, it is a small, monomeric enzyme, which is an advantage for its production in a heterologous host in general [18]. Second, it shows above 90% of maximum β-glucosidase activity over a broad pH range of 6.0–8.5. Finally, it is stable below 30°C and is easy to inactivate rapidly above this temperature. However, the remarkable inhibition of BglMKg β-glucosidase by d-glucose could be a disadvantage for some industrial applications, for example, the enzymatic bioconversion of lignocellulolytic materials. Therefore, we also decided to study the effects of selected metal ions (Table 5) and chemical compounds (Table 6) on the BglMKg β-glucosidase activity.

**Table 5:** The effect of various metal ions on BglMKg β-glucosidase activity

**Ions (5 mM)**	**Relative activity (%)**
Control	100
Mg^2+^	50
Ca^2+^	50
Mn^2+^	52
Ni^2+^	26
Co^2+^	61
Zn^2+^	6

**Table 6:** The effect of selected reagents on BglMKg β-glucosidase activity

**Reagents**	**Relative activity (%)**	
	**10 mM**	**100 mM**
Control	100	100
EDTA	97	81
SDS	< 0.01	< 0.01
Urea	100	95
DTT	110	153
Glutathione oxidized	2	< 0.01
2-mercaptoethanol	98	92
Hydrochloride guanidine	89	58
Tris	41	< 0.01

To examine the possible metal ion requirements of the enzyme (Table 5), activity tests were conducted in MOPS buffer. In the presence of all the ions tested, the β-glucosidase activity was markedly decreased, with the strongest inhibition being found for Zn^2+^ ions. Unfortunately, the negative effect of the presence of metal ions on the BglMKg β-glucosidase activity seems to be another disadvantage for its application in industrial processes. However, the addition of an ethylenediaminetetraacetic acid (EDTA) chelating agent at a low concentration (Table 6) could reduce the effect of the divalent metal ions in this respect.

The β-glucosidase activity of BglMKg was then examined in the presence of selected reagents. Table 6 shows that dithiothreitol (DTT) significantly increases the enzyme activity, whereas sodium dodecyl sulfate (SDS) and oxidized glutathione are both strong inhibitors. The strong inhibition effect of oxidized glutathione and the strong positive effect of DTT on BglMKg activity could suggest the importance of Cys residues in the amino acid sequence of this enzyme. However, it is important to note that the sequence analysis of BglMKg, a member of GH1, showed that the Cys residues are not directly involved in the catalysis (InterProScan, Pfam method). Instead, the analysis of the BglMKg sequence with the DiANNA 1.1 program revealed the possibility of the formation of three putative sulfide bonds in the model structure of the BglMKg enzyme. In this case, the positive effect of the DTT, a strong reducing agent, on BglMKg enzymatic activity would be a result of the prevention of the formation of an intramolecular and/or an intermolecular disulfide bond between the cysteine residues of BglMKg. However, this hypothesis must still be verified empirically. The negative effect of the oxidized glutathione on the studied enzyme activity would be caused by S-glutathionylation of cysteine residues [19].

It should also be noted that the BglMKg enzyme lost enzymatic activity in a 100 mM Tris buffer (Table 6), while changing to a 100 mM sodium phosphate buffer with the same pH restored the activity to 90% of that in the buffer C (a phosphate buffer used in purification procedure, Methods). The inhibition of the enzymatic activity by 2-amino-2-hydroxymethyl-propane-1,3-diol (Tris) was also reported for β-glucosidase from *Agrobacterium faecalis*[20]. In both cases, the inhibition caused by Tris is consistent with that seen for other enzymes such as β-galactosidase [7,21], α-amylase [22], and α-galactosidase [23] and is presumably the reflection of the general affinity of glycosidases for hydroxylated amines [20,24].

## Conclusion

In this study, we sought to identify an enzyme with β-galactosidase activity that would have the capability of hydrolyzing lactose in milk at a low temperature. We constructed a metagenomic library from a sample of Baltic Sea water and identified a gene encoding a novel enzyme with β-galactosidase, β-fucosidase and β-glucosidase activities. The detailed analysis of the data obtained lead us to conclude that the BglMKg enzyme is a bacterial cytosolic β-glucosidase, a new member of GH1 family, characterized by a wide range of enzymatic activities including β-fucosidase and β-galactosidase. It is especially important to note that the enzymatic properties of BglMKg do not fulfill the requirements of a β-galactosidase for commercial use in the dairy industry. However, its wide spectrum of specificity and activity makes this novel, cold-active enzyme potentially interesting for other industrial sectors. We will therefore undertake another study in order to characterize the potential of this novel, cold-adapted β-glucosidase for the hydrolysis of selected, naturally occurring glycosides used in the pharmaceutical, chemical and food industries. GH1 β-glucosidases can also catalyze a reverse transglycosylation reaction [25]. Therefore, we also plan to examine the fitness of BglMkg for this purpose in oligosaccharides synthesis.

## Methods

### Bacterial strains and culture conditions

The *E. coli* LMG 194 (Invitrogen, USA) was used as the host for cloning and expression of the *bglMKg* gene. The *E. coli* strain was grown in Luria-Bertani (LB) medium supplemented with ampicillin (100 μg mL^-1^) at 37°C or 30°C, respectively.

### The metagenomic DNA isolation

The metagenomic DNA was isolated from a sample of Baltic Sea water collected in Kołobrzeg, Poland (54°11^′^N, 15°35^′^E) on 10^th^ January 2009. The seawater temperature was 0.8°C ± 0.2°C that day. The water sample (10 L) was filtered through a cellulose nitrate filter with a pore size of 0.22 μm (Millipore). The material concentrated on the cellulose nitrate membrane was then aseptically transferred to a sterile 50 mL tube and centrifuged at 9,300 × *g* for 30 min at 4°C. Next, the metagenomic DNA was extracted from the pellet using a Genomic Midi kit (A&A Biotechnology, Poland).

### Metagenomic DNA library construction and functional screening

The metagenomic DNA was partially digested using *Bgl*II and *Sal*I endonucleases (Thermo Scientific) and the resulting DNA fragments were purified by precipitation with glycogen (20 mg mL^-1^), sodium acetate (3 M) and isopropanol. The purified DNA fragments were ligated with T4 DNA ligase (Thermo Scientific) into the corresponding sites of the pBAD/*Myc*-HisA plasmid (Invitrogen, USA), and then transformed into *E. coli* LMG194. The recombinant clones were selected on Luria-Bertani agar plates supplemented with ampicillin (0.1 mg mL^-1^), and X-gal (0.02 mg mL^-1^). The agar plates were incubated at 30°C for 18 h and then transferred to 20°C. After incubation at 20°C for 2 days, one colony with presumed β-galactosidase activity turned dark blue because of the hydrolysis of X-gal.

### DNA sequencing and sequence analysis

The metagenomic DNA fragment in the recombinant plasmid pBAD/insMKg isolated from the *E. coli* recombinant clone with β-galactosidase activity was sequenced using a sequencer ABI 3730 xl (Genomed, Poland).

Based on the nucleotide sequence of the metagenomic DNA fragment, the putative ORFs were predicted with the ORF Finder program (http://www.ncbi.nlm.nih.gov/gorf/gorf.html). The DNA sequence homology analyses of the predicted ORFs were conducted with the blastn program (http://www.ncbi.nih.gov/blast). The ORF corresponding to the β-glucosidase/β-galactosidase gene was named *bglMKg*.

The putative promoter sequences in the metagenomic DNA fragment were predicted with the BDGP: Neural Network Promoter Prediction program using the ‘prokaryote’ mode (promoter score cutoff = 0.99; http://www.fruitfly.org/seq_tools/promoter.html), and the BProm program optimized for the identification of bacterial sigma70-dependent promoter sequences, the major *E. coli* promoter class. As described on the BProm website, the program has an *E. coli* promoter recognition accuracy of approximately 80%, and the most recent version is accessible at http://linux1.softberry.com/berry.phtml?topic=bprom&group=programs &subgroup=gfindb.

The amino acid sequence of the BglMKg enzyme was determined with the EMBOSS Transeq application [26]. The molecular weight (M_w_) and isoelectric point (pI) of the BglMKg monomer were calculated using the ExPASy Server tool Compute MW/pI [27].

To define the functional domains and a putative active site, an amino acid sequence analysis was conducted for BglMKg by means of the InterProScan database (http://www.ebi.ac.uk/Tools/pfa/iprscan/). The putative disulfide bonds of BglMKg were predicted with the DiANNA 1.1. online program [28]. The presence and location of the putative signal peptide cleavage site in the BglMKg amino acid sequence was predicted with the SignalP 4.0 server (http://www.cbs.dtu.dk/services/SignalP/) [29]. The BglMKg protein subcellular localization was predicted with the PSORT program (http://psort.hgc.jp/form.html) [30].

### Gene amplification and cloning into a prokaryotic expression system

Based on the *bglMKg* gene sequence, specific primers for PCR were designed (VectorNTI, Invitrogen) and synthesized (Genomed, Poland). The gene was amplified using the forward primer MKg*Bsp*HI 5^′^-AAATC**ATGAAAATAACCTTACCACCCGAATCTCCA**-3^′^ and the reverse primer MKg*Sal*I 5^′^-AAAGTCGAC**TTAACGGGAACCGATTAAGTCACG**-3^′^. Both primers contained recognition sites for the endonucleases *Bsp*HI and *Sal*I. The parts of the primer sequences in boldface are complementary to the nucleotide sequences of the gene, whereas the recognition sites for the restriction endonucleases are underlined and were designed to facilitate cloning. The PCR fragment was cleaned after the enzymatic reaction using a Clean-Up kit (A&A Biotechnology, Poland), then digested with *Bsp*HI and *Sal*I endonucleases, and purified by precipitation. The purified DNA fragments were ligated into the corresponding sites *Nco*I/*Sal*I (Thermo Scientific) of the pBAD/*Myc*-HisA expression vector (pBAD expression system; Invitrogen, USA) under the P_BAD_ promoter, and then transformed into *E. coli* LMG194. This method allowed the expression of the recombinant BglMKg protein with an amino acid sequence identical to the native one.

### Expression and purification of the recombinant enzyme

The *E. coli* LMG194/pBAD/*bglMKg* was grown in LB medium (1 L) containing ampicillin (0.1 mg mL^-1^), and incubated with agitation at 37°C to an OD_600_ of 0.55. The culture was then supplemented with l-arabinose (0.2% w/v) to induce the expression of the *bglMKg* gene and grown for 8 h at 30°C. Next, the *E. coli* cells were harvested by centrifugation at 4°C and 4,600 × *g* for 15 min. The cell pellet was suspended in 30 mL of buffer A (20 mM sodium phosphate buffer, 50 mM NaCl, pH 6.0), and then the *E. coli* cells were disrupted by sonication (6 cycles for 30 s). The cell debris was collected by centrifugation at 13,000 × g for 20 min at 4°C and then the 30 mL of cell-free extract was applied onto an anion exchange Fractogel EMD-DEAE column (Merck) pre-equilibrated with buffer A. The column was washed with buffer A to remove the unbound proteins and the elution was carried out with a linear NaCl gradient in buffer B (50 mM sodium phosphate buffer, 1.5 M NaCl, pH 6.0) at a flow rate of 1 mL min^-1^. Finally, the fractions containing proteins with β-galactosidase activity were collected and dialyzed against buffer C (20 mM sodium phosphate buffer, pH 6.5).

### Estimation of the molecular weight of BglMKg

The molecular weight of the native BglMKg protein was estimated using a gel filtration method. The purified enzyme was applied onto a Superdex 200 HR 10/300 GL gel-filtration column (Amersham Bioscience) pre-equilibrated with 50 mM sodium phosphate buffer, 150 mM NaCl (pH 7.0), and then the protein was eluted with the same buffer at a flow rate of 0.5 mL min^-1^. The following proteins were used for calibration: thyroglobulin (*Mr* = 669,000 Da), apoferritin (*Mr* = 440,000 Da), β-amylase (*Mr* = 200,000 Da), alcohol dehydrogenase (*Mr* = 150,000 Da), bovine serum albumin (*Mr* = 66,000 Da), and carbonic anhydrase (*Mr* = 29,000 Da).

The molecular weight of the denaturated BglMKg protein was estimated using SDS-PAGE [31]. SDS–PAGE was carried out on slabs (10 × 5.5 cm) of 12% polyacrylamide gel. The analyzed samples, before loading on the polyacrylamide gel, were incubated for 5 min at 95°C in the presence of 10% SDS and 0.5% 2-mercaptoethanol.

The protein concentration was determined according to Bradford [32] using BSA as a standard.

### Substrate specificity

The substrate specificity of the purified enzyme was determined at 20°C using 3 mM solutions of the following chromogenic substrates in 20 mM phosphate buffer (pH 6.5): *o*-nitrophenyl-β-d-galactopyranoside (ONPGal), *p*-nitrophenyl-β-d-galactopyranoside, *p*-nitrophenyl-β-d-galacturonide, *p*-nitrophenyl-β-l-arabinopyranoside, *p*-nitrophenyl-β-d-cellobioside,  *p*-nitrophenyl-β-d-mannopyranoside, *p*-nitrophenyl-β-d-glucopyranoside (PNPGlc), *p*-nitrophenyl-α-d-galactopyranoside, *p*-nitrophenyl-β-d-fucopyranoside, *p*-nitrophenyl-β-d-xylopyranoside and *p*-nitrophenyl-β-d-glucuronide. The substrate specificities of BglMKg towards the disaccharides cellobiose, lactose, maltose, trehalose, isomaltose, gentiobiose, nigerose, and sophorose were also determined at 20°C in 20 mM phosphate buffer pH 6.5 (defined as the standard conditions). The final concentration of each disaccharide in the reaction mixture was 30 mM.

### β-Galactosidase activity assay and kinetic parameters

The β-galactosidase activity of BglMKg was assayed by measuring the increase of absorbance at 405 nm from the release of *o*-nitrophenol from ONPGal. The final concentration of ONPGal in the reaction mixture was 3 mM. The enzymatic reaction was carried out at the standard condition, and then the reaction was stopped after 10 minutes with 1 M Na_2_CO_3_. One unit of β-galactosidase activity of BglMKg was defined as the amount of enzyme liberating 1 μM of *o*-nitrophenol per min.

The kinetic parameters of the freshly purified enzyme were determined at 10°C, 20°C and 30°C in 20 mM phosphate buffer with ONPGal (1.0-5.0 mM) or lactose (1.0-5.0 mM) as substrates. The lactose concentration after the enzymatic reaction was determined using a Liquick Cor-Glucose kit (Cormay) to measure the concentration of glucose released during lactose hydrolysis. The *K*_*m*_ and *k*_*cat*_ values were determined from the best fit of the experimental data to the Michaelis*–*Menten equation using non*-*linear regression analysis (GraphPad Prism 5.0)*.*

### β-Glucosidase activity assay and kinetic parameters

The β-glucosidase activity of BglMKg was assayed by measuring the increase of absorbance at 405 nm from the release of *p*-nitrophenol from PNPGlc. The final concentration of PNPGlc in the reaction mixture was 3 mM. The enzymatic reaction was carried out under the standard condition, and then the reaction was stopped after 10 minutes with 1 M Na_2_CO_3_. One unit of β-galactosidase activity of BglMKg was defined as the amount of enzyme liberating 1 μM of *p*-nitrophenol per min._._

The kinetic parameters of the freshly purified enzyme were determined at 10°C, 20°C and 30°C in 20 mM phosphate buffer with PNPGlc (1.0-5.0 mM) or cellobiose (1.0-5.0 mM) as substrates. The cellobiose concentration after the reaction was determined using a Liquick Cor-Glucose kit (Cormay) to measure the concentration of glucose released during cellobiose hydrolysis. The *K*_*m*_ and *k*_*cat*_ values were determined as described above.

### Effect of temperature and pH on β-galactosidase and β-glucosidase activities

The effect of temperature on both enzymatic activities was assayed by incubating the reaction mixtures at temperature ranging from 5°C to 60°C (with 5°C increments) and measuring the activity at the same temperature with the appropriate substrates (ONPGal or PNPGlc at a final concentration of 3 mM) in 20 mM phosphate buffer, pH 6.5.

For thermal stability assays, the purified enzyme was pre-incubated at 10°C, 20°C, 30°C, 40°C and 50°C in the absence of ONPGal or PNPGlc. After incubating for different times (15, 30, 60 and 120 min), both activities were measured by assaying the residual activities at standard conditions (pH 6.5, 20°C).

The optimum pH was determined by assaying the activity of the BgaL enzyme in a 10 mM Britton-Robinson buffer, with pH values ranging from 3.0 to 11.0. The enzymatic activities were quantitated at tested pH value at 20°C with ONPGal or PNPGlc. The enzymatic reactions were stopped after 10 min.

For the pH stability assays, the reaction mixtures with appropriate substrates were incubated at 20°C and pHs ranging from 5.0 to 9.0. After incubating for 15, 30, 60 and 120 min, samples from both reaction mixtures were withdrawn, and the residual enzymatic activities were measured with ONPGal or PNPGlc in 20 mM phosphate buffer pH 6.5 at 20°C. The enzymatic reactions were stopped after 10 min. All the above-presented experiments were performed in triplicate.

### Effects of selected metal ions and reagents on the β-glucosidase activity of BglMKg

The effect of DTT, oxidized glutathione, 2-mercaptoethanol, urea, SDS, Tris, hydrochloride guanidine and EDTA at final concentrations of 10 or 100 mM on BglMKg β-glucosidase activity was assayed under standard reaction conditions. The effect of selected metal ions (Mg^2+^, Ca^2+^, Mn^2+^, Ni^2+^, Co^2+^, Zn^2+^) in a final concentration of 5 mM on BglMKg β-glucosidase activity was determined in 20 mM MOPS buffer at 20°C and pH 6.5.

### Sequence accession numbers

The nucleotide sequence of the *bglMKg* gene has been submitted to the nucleotide sequence database under the accession number HM125785 (GeneBank database). The amino acid sequence of the BglMKg enzyme has been submitted to the protein sequence database under the accession number ADI56259.

## Competing interests

The authors declare that they have no competing interests.

## Authors’ contributions

AWW prepared the metagenomic DNA library, performed the cloning experiments, the protein expression and the purification experiments, characterized the BglMKg enzyme, analyzed the data and was involved in drafting the manuscript. PB participated in the BglMKg protein expression and purification, and characterization. HC coordinated the study, analyzed the data and drafted the manuscript, and JK conceived the study and assisted in its coordination. All the authors read and approved the final manuscript.

## References

[B1] Wong D Marco D Applications of metagenomics for industrial bioproducts. Wong, D. Applications of metagenomics for industrial bioproducts Metagenomics: Theory, methods and applications 2010 1 Norfolk, UK: Caister Academic Press 141 158

[B2] Fernández-Arrojo L Guazzaroni ME López-Cortés N Beloqui A Ferrer M Metagenomic era for biocatalyst identification Curr Opin Biotechnol 2010 21 725 733 10.1016/j.copbio.2010.09.006 20934867

[B3] Lorenz P Eck J Metagenomics and industrial applications Nat Rev Microbiol 2005 3 510 516 10.1038/nrmicro1161 15931168

[B4] Steele HL Jaeger KE Daniel R Streit WR Advances in recovery of novel biocatalysts from metagenomes J Mol Microbiol Biotechnol 2009 16 25 37 10.1159/000142892 18957860

[B5] Sheik Asraf S Gunasekaran P Méndez-Vilas A Current trends of β-galactosidase research and application Current research, technology and education topics in applied microbiology and microbial biotechnology. Volume 2 2010 2 Spain: Formatex 880 890

[B6] Oliveira C Guimaraes PMR Domingues L Recombinant microbial systems for improved β-galactosidase production and biotechnological applications Biotechnol Adv 2011 29 600 609 21514372 10.1016/j.biotechadv.2011.03.008

[B7] Wierzbicka-Woś A Cieśliński H Wanarska M Kozłowska-Tylingo K Hilderbrandt P Kur J A novel cold-adapted β-galactosidase from the *Paracoccus* sp. 32d – gene cloning, purification and characterization Microb Cell Fact 2011 10 108 10.1186/1475-2859-10-108 22166118 PMC3268748

[B8] Turkiewicz M Kur J Białkowska A Cieśliński H Kalinowska H Bielecki S Antarctic marine bacterium *Pseudoalteromonas* sp. 22b as a source of cold-adapted β-galactosidase Biomol Eng 2003 20 317 324 10.1016/S1389-0344(03)00039-X 12919815

[B9] Cieśliński H Kur J Białkowska A Baran I Makowski K Turkiewicz M Cloning, expression and purification of a recombinant cold-adapted β-galactosidase from Antarctic bacterium *Pseudoalteromonas* sp. 22b Prot Expr Purif 2005 39 27 34 10.1016/j.pep.2004.09.002 15596357

[B10] Białkowska AM Cieśliński H Nowakowska KM Kur J Turkiewicz M A new β-galactosidase with a low temperature optimum isolated from the Antarctic *Arthrobacter* sp. 20B: gene cloning, purification and characterization Arch Microbiol 2009 191 825 835 10.1007/s00203-009-0509-4 19771412

[B11] Rodionov DA Yang C Li X Rodionova IA Wang Y Obraztsova AY Zagnitko OP Overbeek R Romine MF Reed S Fredrickson JK Nealson KH Osterman AL Genomic encyclopedia of sugar utilization pathways in the *Shewanella* genus BMC Genomics 2010 11 494 10.1186/1471-2164-11-494 20836887 PMC2996990

[B12] Fang Z Fang W Liu J Hong Y Peng H Zhang X Sun B Xiao Y Cloning and characterization of a β-glucosidase from marine microbial metagenome with excellent glucose tolerance J Microbiol Biotechnol 2010 20 1351 1358 10.4014/jmb.1003.03011 20890102

[B13] Henrissat B Davies GJ Structural and sequence-based classification of glycoside hydrolases Curr Op Struct Biol 1997 7 637 644 10.1016/S0959-440X(97)80072-3 9345621

[B14] Fan HX Miao LL Liu Y Liu HC Liu ZP Gene cloning and characterization of a cold-adapted β-glucosidase belonging to glycosyl hydrolase family 1 from a psychrotolerant bacterium *Micrococcus antarcticus* Enzyme Microb Technol 2011 49 94 99 10.1016/j.enzmictec.2011.03.001 22112277

[B15] Patel GK Kar B Sharma AK Characterization of a thermostable family 1 Glycosyl Hydrolase enzyme from *Putranjiva roxburghii* seeds Appl Biochem Biotechnol 2012 166 523 35 10.1007/s12010-011-9445-2 22086564

[B16] Lauro BD Rossi M Moracci M Characterization of a β-glycosidase from the thermophilic bacterium *Alicyclobacillus acidocardarius* Extremophiles 2006 10 301 310 10.1007/s00792-005-0500-1 16609814

[B17] Suzuki H Okazaki F Kondo A Yoshida KI Genome mining and motif modifications of glycoside hydrolase family 1 members encoded by *Geobacillus kaustophilus* HTA426 provide thermostable 6-phospho-β-glycosidase and β-fucosidase Appl Microbiol Biotechnol 2012 published online 31 May 2012 10.1007/s00253-012-4168-z 22644528

[B18] Hildebrandt P Wanarska M Kur J A new cold-adapted β-d-galactosidase from the Antarctic *Arthrobacter* sp. 32c - gene cloning, overexpression, purification and properties BMC Microbiol 2009 27 9 151 19631003 10.1186/1471-2180-9-151 PMC2723119

[B19] Bilska A Kryczyk A Włodek L The different aspects of the biological role of glutathione Post. Hig. Med. Dosw 2007 61 438 453 17679914

[B20] Kempton JB Withers SG Mechanism of Agrobacterium beta-glucosidase: kinetic studies Biochemistry 1992 31 9961 9969 10.1021/bi00156a015 1390780

[B21] Saishin N Ueta M Wada A Yamamoto I Properties of β-galactosidase purified from *Bifidobacterium longum* subsp. *longum* JCM 7052 grown on gum arabic J Biol Micromol2010a 10 23 31

[B22] Ghalanbor Z Ghaemi N Marashi SA Amanlou M Habibi-Rezaei M Khajeh K Ranjbar B Chan YS Wong JH Ng TB Binding o Tris to *Bacillus licheniformis* alpha-amylase can affect its starch hydrolysis activity Protein Pept Lett 2008 15 212 214 10.2174/092986608783489616 18289113

[B23] Saishin N Ueta M Wada A Yamamoto I Purification and characterization of α-galactosidase I from *Bifidobacterium longum* subsp. *longum* JCM 7052 J Biol Micromol2010b 10 13 22

[B24] Ketudat Cairns JR Esen A β-Glucosidases Cell Mol Life Sci 2010 67 3389 3405 10.1007/s00018-010-0399-2 20490603 PMC11115901

[B25] Sinnott ML Catalytic mechanisms of enzymic glycosyl transfer Chem Rev 1990 90 1171 1202 10.1021/cr00105a006

[B26] Rice P Longden I Bleasby A EMBOSS: the European Molecular Biology Open Software Suit Trends Genet 2000 16 276 277 10.1016/S0168-9525(00)02024-2 10827456

[B27] Gasteiger E Hoogland C Gattiker A Duvaud S Wilkins MR Appel RD Bairoch A Walker JM Protein identification and analysis tools on the ExPASy server The proteomics Protocols Handbook 2005 1 New York: Human Press 571 607

[B28] Ferrè F Clote P DiANNA 1.1: an extension of the DiANNA web server for ternary cysteine classification Nucl Acid Res 2006 34 182 185 10.1093/nar/gkl189 PMC1538812 16844987

[B29] Petersen TN Brunak S von Heijne G Nielsen H SignalP 4.0: discriminating signal peptides from transmembrane regions Nat Methods 2011 8 785 786 10.1038/nmeth.1701 21959131

[B30] Nakai K Horton P PSORT: a program for detecting the sorting signals of proteins and predicting their subcellular localization Trends Biochem Sci 1999 24 34 36 10.1016/S0968-0004(98)01336-X 10087920

[B31] Laemmli UK Cleavage of structural proteins during the assembly of the head of bacteriophage T4 Nature 1970 227 680 685 10.1038/227680a0 5432063

[B32] Bradford MM Rapid and sensitive method for the quantitation of microgram quantities of protein utilizing the principle of protein–dye binding Anal Biochem 1976 72 248 254 10.1016/0003-2697(76)90527-3 942051

